# Bullying experience in urban adolescents: Prevalence and correlations with health-related quality of life and psychological issues

**DOI:** 10.1371/journal.pone.0252459

**Published:** 2021-06-08

**Authors:** Anh Toan Ngo, Long Hoang Nguyen, Anh Kim Dang, Men Thi Hoang, Trang Huyen Thi Nguyen, Giang Thu Vu, Hoa Thi Do, Bach Xuan Tran, Carl A. Latkin, Roger C. M. Ho, Cyrus S. H. Ho

**Affiliations:** 1 Institute for Preventive Medicine and Public Health, Hanoi Medical University, Hanoi, Vietnam; 2 Karolinska Institute, Stockholm, Sweden; 3 Institute for Global Health Innovations, Duy Tan University, Da Nang, Vietnam; 4 Faculty of Medicine, Duy Tan University, Da Nang, Vietnam; 5 Center of Excellence in Pharmacoeconomics and Management, Nguyen Tat Thanh University, Ho Chi Minh City, Vietnam; 6 Institute of Health Economics and Technology, Hanoi, Vietnam; 7 Bloomberg School of Public Health, Johns Hopkins University, Baltimore, Maryland, United States of America; 8 Department of Psychological Medicine, Yong Loo Lin School of Medicine, National University of Singapore, Singapore, Singapore; 9 Institute for Health Innovation and Technology (iHealthtech), National University of Singapore, Singapore, Singapore; 10 Department of Psychological Medicine, National University Health System, Singapore, Singapore; University of Toronto, CANADA

## Abstract

This study examined the 3-month rate of bullying experience, associated factors, and measure the relationships between bullying experience with health-related quality of life and different mental disorders among secondary school students. We performed a cross-sectional study in four secondary schools in Hanoi, Vietnam. Bullying experience was evaluated by using questions about eighteen specific-bullying behaviors. EuroQol-5 dimensions-5 levels (EQ-5D-5L) and Depression, Anxiety, and Stress Scale– 21 items (DASS-21) were used to measure health-related quality of life (HRQOL) and mental health of participants, respectively. Among 712 secondary school students, the 3-month prevalence of physical, social aggression, verbal, and sexual bullying experience were 8.4%; 31.2%; 11.9%, and 2.7%, respectively. Being bullied were negatively associated with levels of classmates and family support, as well as levels of school security. Being overweight or obese was related to a higher likelihood of suffering social aggression compared to normal BMI. Being bullied was significantly associated with the decrement of HRQOL, and the increased risk of depression, anxiety, and stress among adolescents. Findings of this study suggested that holistic approaches involving family, peers, and schools, along with enhancing school security, are potential approaches to reduce the impact of bullying on adolescents’ life and well-being.

## Introduction

Bullying among children is a serious global health problem given its profound physical and psychological consequences on both bullies and victims [1]. Bullying includes aggressive, intentional acts conducted repeatedly and over time against victims to control them, while the victim has limited abilities to cope because of the dominant power of the perpetrator in comparison with the victims [2–4]. Bullying can be in direct forms such as physical attacks (pushing, kicking, hitting) and verbal harassments (name-calling, taunting, or threats, sexual harassment), or in indirect forms such as social aggression (social excluding, rumor spreading, gossiping) [5, 6]. Adolescents and youths are among vulnerable groups to bullying because of their high dependence on others’ care and less autonomy [7]. Moreover, they are at the transition stage where peer influence, which is a major risk factor of bullying, plays a significant role in well-being [7, 8]. Strong evidence has been documented about associations between bullying victimization among adolescents and various social and health problems such as mental disorders, non-suicidal self-injury, suicidal ideation, and suicide behaviours [1, 9, 10]. Moreover, both victims and bullies suffered from the deterioration of academic performance, prosocial skills, mental well-being, and life satisfaction [11–13]. These harms of different bullying types pose great challenges for school-based health promotion strategies.

The prevalence of bullying victimization is diverse across nations. A multi-country cross-sectional study showed that the rate of bullying ranged from 2.4% to 31.9% among boys and from 1.5% to 34.4% among girls [14]. Physical behaviours were reported at low (below 7%) in Ethiopia, Peru, and Vietnam, while being punched/kicked/beaten up and being hurt physically in other ways were reported at 17.3% and 12.2% among children in India, respectively [14]. In China, there were 26.10%, 9.03%, and 28.90% children experiencing bullying, being bullied, and witnessing bullying, respectively [10]. Another study examined the pooled prevalence of bullying victimization of children reported 30.5% (95% CI: 30.2–31.0%) of children [15]. The prevalence and forms of bullying differ by gender and age groups. Boys were more likely to involve in bullying acts than girls [16]. The prevalence of bullying is the highest among adolescents in secondary schools, and diminish among students in high schools [17]. People with disabilities, ethnic minorities or suffering from obesity have a greater chance of experiencing bullying compared to other peers [18, 19]. Besides, parents, friends, and school’s support are positively related to a lower risk of bullying victimization because they can help children to early detect relationship problems and assist problem-solving [15, 20].

In Vietnam, a previous study suggested that approximately one-third of students at secondary and high schools experienced as victims, bullies, or bully-victims at both times [21]. Positive associations between bullying victimization and depression was also found [21]. However, the residential area was not taken into account when examining the bullying experience among children in the study, in which violent behaviour was proved to be more prevalent in the metropolis [22, 23]. In addition, evaluating the quality of life and mental disorders of children undergoing bullying plays a vital part to identify manners to minimize negative influences of bullying on children’s well-being [24]. This study aimed to examine the 3-month rate of bullying experience, identify associated factors, and measure the relationships between bullying experience with health-related quality of life and different mental problems among students at secondary schools in Hanoi, a metropolitan area of Vietnam.

## Methods

### Participants

From January to September 2020, we performed a cross-sectional study at four secondary schools in Hanoi, a metropolis of Vietnam. We recruited students who met the following inclusion criteria: 1) aged from 11 to 14 (corresponding to 6^th^ to 9^th^ grade); 2) studying in selected schools and 3) accepting to be the study participant. Informed consents were given to the principals of selected schools, teachers, students, and their parents/guardians before collecting data.

The sample size was calculated by using the formula to estimate a population proportion with specified relative precision. In this study, we used confidence level (%) α = 0.05, expected population proportion p = 47.5% [25], relative precision ɛ = 0.15, the sample size was 189 students per school, resulting in a total of 756 students being recruited to participate in the study. A multi-stage sampling technique was applied. First, we randomly selected four secondary schools in urban settings of Hanoi, Vietnam. Then, in each school, we randomly selected two classes per grade, resulting in 8 classes per school and 32 classes in four schools with a total of approximately 1200 students. We then developed the list of students in all classes and randomly selected 756 students by computer software. After screening the data, the dataset of 712 students was used for final analysis (completion rate = 94.2%).

### Procedure

In this study, we anonymously collected students’ data by using a self-administered structured questionnaire. Given the sensitive content of the question, this questionnaire was developed under the instruction of experts in child maltreatment and bullying. The questionnaire was carefully piloted among children and adolescents to ensure that the content of the questions was understandable and logical. We also revised all texts and language errors in the questionnaire. The final version of the questionnaire was approved by the principals of selected schools. Before administration, the principal investigator briefly introduced the purposes of the study and guided students in filling the questionnaire. In each class, the questionnaire was distributed to the students during non-teaching hours and then collected by the researcher. Students spent 15 to 20 minutes to complete the survey.

### Instrumentation

#### Socio-demographic characteristics

We included specific questions to measure socio-demographic characteristics including age, gender (male/female), type of family (nuclear/others), height, and weight. We then calculated the body mass index by using height and weight information (body mass index (kg/m^2^) = height/weight^2^). Students were classified into three groups: underweight (< 18.5 kg/m^2^), normal (18.5- < 25.0 kg/m^2^) and overweight/obesity (≥25.0 kg/m^2^).

#### Bullying experiences

To identify the bullying experience among students, we asked them a series of multiple-choice questions about their experience of following bullying acts in the last three months before the survey. These acts were selected based on the definition of different types of bullying in the Children Law in Vietnam [26]:

1. In the last three months, have you ever been:
  - Experienced physical violence (slapping, hitting, kicking or beating)
  - Threatened with a weapon (e.g. scissors, knife or a gun)
  - Locked in the classroom, toilet or other rooms
  - Robbed money
  - None of them
2. In the last three months, have someone ever
  - Disrespected and refused to listen to your opinion
  - Taken control by someone and made you do what they want?
  - Boycotted by someone or incited others to boycott or isolate you?
  - Bullied or scared you?
  - Forced or seduced you to participate in destructive and anti-social behaviours?
  - Put pressure on you to achieve results beyond your capabilities?
  - None of them
3. In the last three months, have someone ever
  - Talked profanely or looked down on yourself and my family by someone, or made fun of, mimicked, or imitated your behaviours?
  - Commented or told a story in order to insult or offend / ridicule you?
  - Discussed, gossiped, or spread bad rumours about you
  - Pretended to be you on the internet and put private information about you online?
  - None of them
4. In the last three months, have someone ever:
  - Pulled, taken off, tugged your skirt / shirt / pants?
  - Touched a sensitive part of your body?
  - Forced you to have sex?
  - Sent text messages or emails asking you to have sex that you did not want?
  - None of them

Participants selected any options of the above questions were categorized as “Having experience”. For question 1, we aimed to investigate the physical bullying victimization, while for questions 2, 3, 4, we aimed to examine the social aggression, verbal bullying, and sexual bullying experience, respectively. Students who did not select any of them or select “None of them” were classified as the “No experience”.

#### Family, classmates, and teachers support

Students were asked seven self-reported questions about perceived levels of support from family (item 1 and 2), classmates (item 3 and 4), and teachers (item 5, 6, and 7). This approach has been used previously [27]:

1. My parents do not understand me or care about my feelings.
2. My parents do not listen to me or not pay attention to the problems I have.
3. My classmates are very friendly.
4. Classmates respect me and listen to my opinion.
5. My teachers help me when I’m sad or having problems.
6. My teachers take care of me and support me in achieving the best results.
7. My teachers respect myself and listen to me.

There were five levels of response for each statement: 1 = Totally disagree; 2 = Slightly agree; 3 = Somewhat agree; 4 = Mostly agree, and 5 = Totally agree. We reversed scores of questions 1 and 2 when calculating the total score. The total score of each domain was calculated by summing scores of all items in the domain and divided by the number of items in each domain. The score ranged from 1 to 5, which a higher score indicated higher levels of support. Cronbach’s alpha of these items was 0.820, suggesting acceptable internal consistency reliability. In addition, we asked students to rate levels of school security with an 11-point scale from 0 “Complete unsafety” to 10 “Complete safety”.

#### Health-related quality of life (HRQOL)

HRQOL of students was assessed by utilizing the Vietnamese version of EuroQol-5 dimensions-5 levels (EQ-5D-5L) [28]. This scale evaluated the HRQOL of respondents in five questions, which were corresponding to dimensions: mobility, usual activity, self-care, pain/discomfort, and anxiety/depression. Each question had five response options from No problem to Extreme problem. The combination of responses of five questions produces a health state. By using the crosswalk value set of the Vietnamese population [28], we converted each health state to its corresponding EQ-5D index. A higher EQ-5D index meant a higher HRQOL. The Cronbach’s alpha of this instrument was 0.6051.

#### Mental problems

The Depression, Anxiety, and Stress Scale—21 Items (DASS-21) was used to evaluate the depression, anxiety, and stress conditions of students in the last seven days [29]. This scale has been validated in Vietnamese adolescents previously [29]. Each item of this scale had three options: 0 = Did not apply to me at all; 1 = Applied to me to some degree, or some of the time; 2 = Applied to me to a considerable degree or a good part of the time; and 3 = Applied to me very much or most of the time. Students were classified to have depressive symptoms, anxiety, and stress if they had ≥ 10 points for depression-related questions, ≥ 8 points for anxiety-related questions, and ≥ 15 points for stress-related questions [29]. The Cronbach’s alphas for depression, anxiety, and stress in this study were 0.780; 0.602, and 0.730.

### Data analysis

Data analysis was conducted using the Stata 15.0 software. P-value < 0.05 was used to detect statistical significance. Missing data were handled using the listwise deletion method. Variance inflation factor (VIF) was tested to examine the collinearity in regression models.

The 3-month prevalence of different types of bullying (physical bullying, social aggression, verbal bullying, and sexual bullying) were described in general and according to different socio-demographic characteristics. We also defined “poly-victimization” as students who suffered from two or more forms of bullying [30]. The Chi-squared test was used to examine the difference in the 3-month prevalence of bullying victimization regarding different groups. Associated factors with each form of bullying victimization were identified by using the multivariate Logistic regression model. Dependent variables in the model were physical bullying, social aggression, verbal bullying, and sexual bullying experiences (Victims/Non-victims), while the independent variables were prior-defined-sociodemographic characteristics, levels of school security and levels of parents, classmates, and teacher support.

EQ-5D index and the rate of depression, anxiety, and stress among students were described according to types of bullying victimization. The difference between victims and non-victims was examined using the Mann-Whitney test (for EQ-5D index) and the Chi-squared test (for the rate of depression, anxiety, and stress). Associations between the EQ-5D index and types of bullying victimization as well as the number of forms of bullying experienced were identified by using the multivariate Tobit regression. Meanwhile, relationships between depression, anxiety, and stress with types of bullying victimization as well as the number of bullying forms experienced, were determined by using the multivariate Logistic regression. These models were adjusted for prior-defined-sociodemographic characteristics, levels of school security, and levels of parents, classmates, and teacher support.

### Ethical approval

Given that bullying experience was the sensitive information, before implementing the survey, we contacted and send an information package to the principals of selected schools, teachers, students, and their parents/guardians. Each package contained information about the study purposes, designs, inclusion criteria, and potential benefits and harms when participating in the study. We also underlined that students’ participation was not mandate, and they could withdraw to the study anytime without any influences on the current relationships between them and schools. They could also skip questions that they did not want to answer. We did not collect any individual data for confidentiality; hence, re-identifying students’ identifications is impossible. We also offered the hotline in the package if students needed helps. Both students and their parents/guardians signed into the written informed consent. The institutional review board of the Hanoi Medical University granted the study protocol (Code: 22NCS17/HDDDDHYHN).

## Results

Among 712 secondary school students participating in the study, there were 414 female students and 298 male students. The 3-month prevalence of physical, social aggression, verbal, and sexual bullying experiences were 8.4%, 31.2%, 11.9%, and 2.7%, respectively. The 3-month prevalence of being physically bullied among children aged 12 and 13 years old was two times higher than that in those aged 11 and 14 (p<0.05). The 3-month prevalence of emotional bullying experience was different among age groups and body mass index groups (p<0.05). Male students (4.4%) had approximately three times higher in the rate of sexual harassment compared to female counterparts (1.5%) (p<0.05) **(Table 1)**.

**Table 1:** Demographic characteristics of respondents.

Characteristics	Total	Physical bullying		Social aggression		Verbal bullying		Sexual bullying	
		n (%)	p-value	n (%)	p-value	n (%)	p-value	n (%)	p-value
Total	712	60 (8.4)		222 (31.2)		85 (11.9)		19 (2.7)	
Gender									
Male	298	31 (10.4)	0.11	85 (28.5)	0.19	36 (12.1)	0.92	13 (4.4)	0.02
Female	414	29 (7.0)		137 (33.1)		49 (11.8)		6 (1.5)	
Age (years old)									
11	216	12 (5.6)	0.03	46 (21.3)	<0.01	20 (9.3)	0.08	9 (4.2)	0.43
12	107	13 (12.2)		24 (22.4)		8 (7.5)		2 (1.9)	
13	211	25 (11.9)		82 (38.9)		33 (15.6)		4 (1.9)	
14	178	10 (5.6)		70 (39.3)		24 (13.5)		4 (2.3)	
Type of family									
Nuclear	461	41 (8.9)	0.54	147 (31.9)	0.58	49 (10.6)	0.14	15 (3.3)	0.19
Others	251	19 (7.6)		75 (29.9)		36 (14.3)		4 (1.6)	
Body mass index groups									
Normal	307	22 (7.2)	0.45	86 (28.0)	0.03	33 (10.8)	0.18	6 (2.0)	0.28
Underweight	368	34 (9.2)		118 (32.1)		43 (11.7)		13 (3.5)	
Overweight and obesity	32	4 (12.5)		16 (50.0)		7 (21.9)		0 (0.0)	

Slapping, hitting, kicking, or beating were the most common behaviour of physically bullying (8.2%) **(Table 2)**. Meanwhile, the most common behaviour of social aggression was “Someone disrespects and refuses to listen to my opinion” (24.0%), following by “Takes control and makes me do what they want” (10.0%). “Talks profanely looks down on me, my family, makes fun of, mimics or imitates my behaviour” was the most frequent verbal bullying act that students suffered from (7.7%), following “Discusses, gossip, or spread bad rumours about me” (6.5%). Finally, “Touches a sensitive part of my body” (2.1%) and “Pulls, takes off, tugs skirt/shirt/pants” (1.5%) were the two most common acts of sexual bullying that the students experienced in the last three months. Overall, 24.4% of students suffered from one type of bullying, 9.7% suffered from two types, and 3.4% experienced three types or more.

**Table 2:** Types of bullying in the last three months.

Type of bullying	Male	Female	Total	p-value
	n (%)	n (%)	n (%)	
**Physical bullying**				
Being acted physical violence (slapping, hitting, kicking or beating)	31 (10.4)	27 (6.5)	58 (8.2)	0.06
Threatens with a weapon (e.g. scissors, knife or a gun)	3 (1.0)	1 (0.2)	4 (0.6)	0.18
Being locked in the classroom, toilet or other rooms	2 (0.7)	1 (0.2)	3 (0.4)	0.38
Being robbed money	3 (1.0)	1 (0.2)	4 (0.6)	0.18
**Social aggression**				
Someone disrespects and refuses to listen to my opinion	66 (22.2)	105 (25.4)	171 (24.0)	0.32
Takes control and makes me do what they want	31 (10.4)	40 (9.7)	71 (10.0)	0.75
Boycotts or incites others to boycott or isolate me	13 (4.4)	30 (7.3)	43 (6.0)	0.11
Bullies or creates an atmosphere that makes me scared	15 (5.0)	16 (3.9)	31 (4.4)	0.45
Forces or seduces me to participate in destructive and anti-social behavior	7 (2.4)	8 (1.9)	15 (2.1)	0.70
Put pressure to achieve results beyond my capabilities	39 (13.1)	46 (11.1)	85 (11.9)	0.42
**Verbal bullying**				
Talks profanely, looks down on me, my family, makes fun of, mimics, imitates my behavior	28 (9.4)	27 (6.5)	55 (7.7)	0.16
Comment or tell a story in order to insult or offend / ridicule me	16 (5.4)	22 (5.3)	38 (5.3)	0.97
Discusses, gossip, or spread bad rumors about me	13 (4.4)	33 (8.0)	46 (6.5)	0.05
Pretends to be me on the internet and puts private information about me online	11 (3.7)	7 (1.7)	18 (2.5)	0.09
**Sexual bullying**				
Pulls, takes off, tugs skirt / shirt / pants	9 (3.0)	2 (0.5)	11 (1.5)	<0.01
Touches a sensitive part of my body	10 (3.4)	5 (1.2)	15 (2.1)	0.049
Being forced to have sex	6 (2.0)	3 (0.7)	9 (1.3)	0.13
Sends text messages or emails asking me to have sex that I don’t want	6 (2.0)	3 (0.7)	9 (1.3)	0.13
**Number of types of bullying experienced**				
0	191 (64.1)	254 (61.4)	445 (62.5)	0.22
1	65 (21.8)	109 (26.3)	174 (24.4)	
2	28 (9.4)	41 (9.9)	69 (9.7)	
≥ 3	14 (4.7)	10 (2.4)	24 (3.4)	

**Table 3** indicates that students who had physical bullying, social aggression, and verbal bullying experience perceived significantly lower levels of support from family, classmates, and teachers and school security compared to non-victims (p<0.05). For sexually bullying, only levels of support from friends were differently perceived between those with and without experience (p<0.05).

**Table 3:** Type of bullying victimization and supports from family, classmates, and teachers.

Type of bullying victimization	Support from family (1–5)		Support from classmates (1–5)		Support from teachers (1–5)		Perceived levels of school security (0–10)	
	Mean (SD)	p-value	Mean (SD)	p-value	Mean (SD)	p-value	Mean (SD)	p-value
Physical bullying								
No	4.0 (1.0)	<0.01	4.0 (1.0)	<0.01	4.2 (1.1)	<0.01	8.4 (1.9)	0.02
Yes	3.5 (1.2)		3.4 (1.2)		3.6 (1.3)		7.6 (2.3)	
Social aggression								
No	4.2 (1.0)	<0.01	4.1 (1.0)	<0.01	4.3 (1.0)	<0.01	8.7 (1.8)	<0.01
Yes	3.5 (1.1)		3.8 (1.1)		3.8 (1.2)		7.7 (2.2)	
Verbal bullying								
No	4.0 (1.0)	<0.01	4.0 (1.0)	<0.01	4.2 (1.1)	0.02	8.4 (1.9)	<0.01
Yes	3.4 (1.2)		3.5 (1.1)		3.8 (1.3)		7.6 (2.5)	
Sexual bullying								
No	4.0 (1.0)	0.08	4.0 (1.0)	0.02	4.1 (1.1)	0.43	8.4 (1.9)	0.36
Yes	3.3 (1.5)		3.3 (1.2)		3.7 (1.6)		7.1 (3.7)	

The results of the multivariate analysis are shown in **Table 4**. Students receiving higher levels of support from classmates (OR = 0.67, 95%CI = 0.48–0.94) and family (OR = 0.70, 95%CI = 0.52–0.95) had a lower likelihood of being physically bullied. These associations were also found in verbal bullying experience when a higher level of support from classmates (OR = 0.63, 95%CI = 0.48–0.84) and family (OR = 0.60; 95%CI = 0.47–0.76) were negatively associated with being verbally bullied. Having a higher level of support from family was related to a lower odd of having social aggression experience. Meanwhile, students who were overweight or obese were more likely to suffer social aggression (OR = 3.35; 95%CI = 1.12–10.08) compared to those with normal BMI. Females had a lower likelihood of having sexually bullying experience compared to male students. The sexual bullying experience was also lower in students aged 13 and 14 years old compared to those aged 11 years old.

**Table 4:** Associated factors with bullying victimization.

Characteristics	Physical bullying		Social aggression		Verbal bullying		Sexual bullying	
	OR	95%CI	OR	95%CI	OR	95%CI	OR	95%CI
Gender (Female vs Male-reference)								
Male	REF		REF		REF		REF	
Female	0.74	0.38–1.45	1.34	0.88–2.03	0.97	0.56–1.66	0.29**	0.10–0.87
Age								
11 years old	REF		REF		REF		REF	
12 years old	1.95	0.68–5.61	0.95	0.45–2.04	0.80	0.29–2.25	0.49	0.09–2.67
13 years old	1.17	0.49–2.80	1.42	0.82–2.45	1.00	0.48–2.06	0.24**	0.06–0.99
14 years old	0.37*	0.12–1.10	1.49	0.86–2.59	0.76	0.36–1.59	0.23**	0.05–0.97
Type of family								
Nuclear	REF		REF		REF		REF	
Other	0.99	0.49–1.99	0.98	0.64–1.50	1.68*	0.97–2.91	0.34	0.09–1.33
Body mass index group								
Normal	REF		REF		REF		REF	
Underweight	0.99	0.49–2.00	0.82	0.54–1.25	1.09	0.62–1.89	0.61	0.20–1.87
Overweight and obesity	2.46	0.62–9.80	3.35**	1.12–10.08	0.72	0.15–3.52		
Support from family (per score)	0.70**	0.52–0.95	0.58***	0.48–0.71	0.60***	0.47–0.76	0.71	0.47–1.08
Support from classmates (per score)	0.67**	0.48–0.94	0.90	0.72–1.13	0.63***	0.48–0.84	0.72	0.45–1.15
Support from teachers (per score)	0.93	0.67–1.30	0.92	0.74–1.15	1.12	0.84–1.48	0.94	0.58–1.54
Perceived level of security in school (per score)	0.95	0.81–1.13	0.87**	0.78–0.98	0.90	0.79–1.04	0.86	0.69–1.07

* p<0.*1;*

** p<0.*05;*

***p<0.*01*.

When examining the difference of HRQOL, depression, anxiety, and stress between those with and without bullying experience, **Table 5** shows that students having bullying experience had lower EQ-5D index, higher rates of depression, anxiety, and stress compared to those without experience (p<0.05), except for the anxiety rate among those with and without sexual bullying experience.

**Table 5:** Health-related quality of life and Depression, Anxiety and Stress regarding Types of Bullying victimization.

Type of bullying victimization	EQ-5D index		Depression		Anxiety		Stress	
	Mean (SD)	p-value	n (%)	p-value	n (%)	p-value	n (%)	p-value
Total	0.89 (0.11)		223 (31.3)		392 (55.1)		278 (39.0)	
Physical bullying								
No	0.90 (0.11)	<0.01	28 (5.7)	<0.01	21 (6.6)	0.11	19 (4.4)	<0.01
Yes	0.83 (0.14)		32 (14.4)		39 (10.0)		41 (14.8)	
Social aggression								
No	0.91 (0.10)	<0.01	120 (24.5)	<0.01	66 (20.6)	<0.01	100 (23.0)	<0.01
Yes	0.86 (0.12)		102 (45.7)		156 (39.8)		122 (43.9)	
Verbal bullying								
No	0.90 (0.10)	<0.01	42 (8.6)	<0.01	24 (7.5)	<0.01	34 (7.8)	<0.01
Yes	0.84 (0.15)		43 (19.3)		61 (15.6)		51 (18.4)	
Sexual bullying								
No	0.89 (0.11)	0.02	8 (1.6)	0.01	5 (1.6)	0.10	6 (1.4)	<0.01
Yes	0.83 (0.19)		11 (4.9)		14 (3.6)		13 (4.7)	

**Table 6** depicts the associations between bullying experience, HRQOL, and different mental problems. After adjusting to other covariates, students suffering from physical bullying (Coef. = -0.05; 95%C = -0.08 - -0.02), social aggression (Coef. = -0.02; 95%C = -0.04 - -0.01) and verbal bullying (Coef. = -0.03; 95%C = -0.06 - -0.01) had significantly lower HRQOL compared to non-victims. All of the bullying forms were found to be associated with higher odds of stress. Students experiencing social aggression and sexual bullying had a higher likelihood of developing depression. Meanwhile, students experiencing social aggression were more likely to suffer anxiety compared to those without experience (OR = 1.73; 95%CI = 1.13–2.66).

**Table 6:** Associations between bullying victimization and HRQOL and mental health impairment.

Type of bullying victimization	EQ-5D index^¶^		Depression^¶^		Anxiety^¶^		Stress^¶^	
	Coef.	95%CI	OR	95%CI	OR	95%CI	OR	95%CI
Physical bullying (Yes vs No-ref)	-0.05*	-0.08; -0.02	1.96	0.94; 4.08	1.30	0.63; 2.68	2.95*	1.45; 5.99
Social aggression (Yes vs No-ref)	-0.02*	-0.04’ -0.01	1.61*	1.03; 2.53	1.73*	1.13; 2.66	2.09*	1.38; 3.18
Verbal bullying (Yes vs No-ref)	-0.03*	-0.06; -0.01	1.35	0.75; 2.42	1.46	0.81; 2.64	1.99*	1.14; 3.49
Sexual bullying (Yes vs No-ref)	-0.04	-0.09; 0.00	4.01*	1.23; 13.06	2.17	0.67; 6.98	3.98*	1.29; 12.2
Number of types of bullying experienced (vs None-ref)								
1	-0.01	-0.03; 0.01	1.75*	1.06; 2.89	1.62*	1.02; 2.58	2.00*	1.26; 3.17
2	-0.04*	-0.07; -0.02	1.02	0.51; 2.01	2.52*	1.29; 4.94	2.85*	1.52; 5.31
≥ 3	-0.08*	-0.13; -0.04	10.33*	2.62; 40.76	1.33	0.45; 3.92	6.29*	2.04; 19.44

^*¶*^ *The model was adjusted for gender*, *age*, *type of family*, *body mass index*, *support from family/classmates/teachers*, *and perceived level of security in school*.

* p<0.*05*.

## Discussion

Our findings showed that the most common form of bullying among adolescents in urban settings in Vietnam was social aggression, following by verbal bullying, physical bullying, and sexual bullying. This study also showed negative relationships between being bullied and adolescents’ quality of life and mental health, as well as the active role of family-friends-school support in bullying prevention.

Our study echoed the previous findings that bullying experience was a widespread phenomenon among adolescents in the metropolitan area of Vietnam [21, 31]. In our study, more than one-third (37.5%) of adolescents were involved in at least one type of bullying in the last three months. This result was slightly lower than previous work in Vietnam, which indicated that 44.7% of students were involved in life-time bullying victimization [32]. Our result was three times higher than findings from a cross-national survey in 40 countries in 2005/2006 among adolescents aged 11, 13, and 15 years old (16.6% had life-time experience of victims, 8.6% to 45.2% in boys and 4.8% to 35.8% in girls) [33]. Other studies in India found that the prevalence of bullying victims among urban school students ranged from 29.7% to 61% [34, 35]. Notably, in this current study, we found that 13.1% of adolescents suffered from poly-victimization. This finding was similar to a prior study among children and adolescents in the United States (10%) [36], but significantly lower than that in a previous study among high school students in Hanoi, Vietnam (31%) [31]. There were several reasons which can be used to explain the discrepancies among studies. First, differences in screening tools, cut-off thresholds, and recall period for bullying experiences across countries lead to differences in the prevalence. For example, the study in Vietnam used the Juvenile Victimisation Questionnaire (JVQ) Revised 2 toolkit with 34 behaviours [31], or another study asked participants to report bullying behaviours in children’s life-time [21]. Previous research in India used four questions to screen victims of violence, combining the Peer Interaction in Primary Schools Questionnaire instrument to examine the level of bullying perpetrators or victims of violence [34]. In this study, we used a pool of eighteen specific-questions about bullying behaviours since the content of questions in standardized measures such as JVQ might not be appropriate in the context of secondary school. Moreover, we limited the recall period to the last three months before the survey to minimize recall bias.

Social aggression was the most common type of bullying among students in urban secondary schools, following by physical, verbal, and sexual bullying. This result was different in findings from studies in other countries such as India, where “teasing, making fun, taking things, and making feels sad” were the most common bullying acts [34, 35]. Indeed, in recent years, Vietnam has implemented some campaigns to prevent bullying and mitigate its impacts on children’s well-being; therefore, the rates of direct bullying such as physical or sexual violence were low in this study. However, non-physical violence behaviours such as verbal violence or social isolation are difficult to recognize and prevent. A study by Guerra et al. showed a significant shift in patterns of violence by age [37]. In the elementary school, violence is usually in physical forms; whereas at a higher education level like middle and high schools, adolescents tend to suffer from bullying problems related to being under pressure from others or being jealous and gossiped. In other words, along with increased age and acquired knowledge, the prevalent form of violence also changes from direct to indirect forms. This needs to be considered in the development of robust intervention programs to prevent bullying in the students [37].

Our multivariate results reaffirmed the negative relation between age and a likelihood of being bullied. During the transition time from primary to secondary school (e.g. sixth and seventh grades), students may be more likely to find social dominance to prove their power over their peers, which might put them at a high risk of being bullied, especially physical and sexual bullying [32, 38]. Notably, we found that females were less likely to suffer from sexual bullying than their male counterparts. This result was contrary to the common misconception in the community that females were at higher risk of sexual harassment compared to males [39, 40]. This phenomenon could be explained by the fact that in our study, the rate of “pulls, takes off, tugs skirt / shirt / pants” acts among male children were significantly higher than female children. We believe this may be related to the matter that, according to our observations, male students practiced pantsing as a prank in school, which has been also recorded as a pervasive behavior in previous literature [41]. Children might not yet realize that this behaviour was considered sexual harassment. This suggests the need to design health education programs to raise awareness of students, especially boys, of such sexual harassment behaviours.

Overweight and obesity students were more likely to experience social aggression. In prior literature, overweight and obese youths and adolescents were also found to be associated with bullying victimization [42]. In this study, we specified that these people were at risk of social aggression, which might be related to body image perception. A prior study among Danish adolescents showed that body image was a good mediator to explain the U-shape association between overweight/obesity and being bullied [43]. More studies should be investigated the pathway of this relationship.

Our findings were in line with the previous studies to show that positive parent-child, friend-friend, and teacher-student relationships were the significant protective factors from being bullied [44–46]. Previous studies indicated that students who lacked teacher and peer supports were more likely to be bullying victims. In other words, bullying victims might be less likely to occur if the teachers knew the situations [47], as well as if peers did not accept bullying behaviours [48]. However, in a previous study in Vietnam, the authors found no association between parent support and bullying victimization, and they argued that students in their sample might not discuss the bullying problems with their parents [32]. Further studies should determine the reasons for this phenomenon.

The findings of this study contributed to the current literature about the effects of different types of bullying on students’ HRQOL and mental health. Prior studies showed that having bullying victimization experience increased significantly the risk of depression as well as suicidal ideation [21, 49, 50]. In this study, regarding HRQOL, we found that physical bullying had the strongest influence compared to other types of bullying, which might be because this type of bullying affects at least four (mobility, self-care, usual activity, and pain/discomfort) out of five dimensions of the EQ-5D-5L instrument. We did not find the decrement of HRQOL between those with and without sexual bullying experience, which might be due to the small sample size of this group. Meanwhile, sexual bullying contributed to the highest variation in depression and stress compared to other bullying types, suggesting the magnificent effect of sexual harassment on the mental health of adolescents.

### Limitations

Our study has some limitations. Firstly, although the study used a multi-stage sampling method and involved a simple random sampling approach to select study participants, our results may not reflect fully the bullying phenomenon among adolescents in urban Viet Nam. In addition, our study did not include adolescents who were out-of-school, not at school during the study period, or in private schools. Second, our study used a cross-sectional design, which was unable to develop causal relationships between bullying and associated factors, as well as relationships with mental health status and quality of life. Third, we did not investigate the prevalence of bullying perpetrators among our sample given sensitive information. Moreover, this study did not investigate the positive and negative influence of social media, as well as accessibility of different programs in preventing school bullying in the community, which might be important to predict the bullying experience in the adolescents. Finally, our study asked participants about the bullying experiences over the past 3 months, which may lead to recall bias as well as miss any events occurring later than 3 months. We did not used a validated instrument to measure bullying experience, which might reduce our comparability to other studies. Further studies with holistic approaches should be warranted.

## Conclusions

This study indicated a high 3-month prevalence of bullying experience, especially social aggression, among students in metropolitan secondary schools in Vietnam, as well as the decrement of HRQOL and high risks of mental problems in bullying sufferers. Holistic approaches involving family, peers, and schools, along with enhancing school security, are potential methods to reduce the impact of bullying on adolescents’ life and well-being.
